# Generation of gene-corrected functional osteoclasts from osteopetrotic induced pluripotent stem cells

**DOI:** 10.1186/s13287-020-01701-y

**Published:** 2020-05-15

**Authors:** Xiaojie Xian, Roksana Moraghebi, Henrik Löfvall, Anders Fasth, Kim Henriksen, Johan Richter, Niels-Bjarne Woods, Ilana Moscatelli

**Affiliations:** 1 Department of Molecular Medicine and Gene Therapy, Lund Strategic Center for Stem Cell Biology, Lund University, BMC A12, 221 84 Lund, Sweden; 2 Nordic Bioscience, Herlev, Denmark; 3 Department of Pediatrics, Institute of Clinical Sciences, University of Gothenburg, Gothenburg, Sweden

**Keywords:** Infantile malignant osteopetrosis, iPSC generation, Gene transfer, Osteoclast differentiation

## Abstract

**Background:**

Infantile malignant osteopetrosis (IMO) is an autosomal recessive disorder characterized by non-functional osteoclasts and a fatal outcome early in childhood. About 50% of patients have mutations in the *TCIRG1* gene.

**Methods:**

IMO iPSCs were generated from a patient carrying a homozygous c.11279G>A (IVS18+1) mutation in *TCIRG1* and transduced with a lentiviral vector expressing human *TCIRG1*. Embryoid bodies were generated and differentiated into monocytes. Non-adherent cells were harvested and further differentiated into osteoclasts on bovine bone slices.

**Results:**

Release of the bone resorption biomarker CTX-I into the media of gene-corrected osteoclasts was 5-fold higher than that of the uncorrected osteoclasts and 35% of that of control osteoclasts. Bone resorption potential was confirmed by the presence of pits on the bones cultured with gene-corrected osteoclasts, absent in the uncorrected IMO osteoclasts.

**Conclusions:**

The disease phenotype was partially corrected in vitro, providing a valuable resource for therapy development for this form of severe osteopetrosis.

## Background

Infantile malignant osteopetrosis (IMO) is the most severe form of the genetically heterogeneous group of disorders named osteopetrosis [1]. IMO is characterized by defective functionality or differentiation of osteoclasts that leads to increased bone mass, dental abnormalities, and frequent bone fractures [2]. IMO patients also display a progressive reduction of marrow cavities leading to anemia, hepatosplenomegaly, and frequent infections [3, 4]. Furthermore, compression of the cranial nerves leads to impairment of neurologic functions with blindness and sometimes also deafness [3, 4]. More than 50% of the patients have mutations in the *TCIRG1* gene encoding the a3 subunit of the V-ATPase proton pump, which is necessary for the acidification of the resorption lacunae [5]. The only curative treatment to date for IMO is allogeneic hematopoietic stem cell transplantation (HSCT), and IMO is usually fatal within the first 10 years of life if not treated [1]. Despite the greatly improved outcome overtime of HSCT for IMO [2, 6, 7], alternative autologous therapies such as HSC-targeted gene therapy [8, 9] have the potential to restore the resorptive function of osteoclasts without some of the complications associated with allogeneic HSCT such as limited availability of a matching donor and graft-versus-host disease [10].

Patient-derived induced pluripotent stem cells (iPSCs) provide a valuable resource for pathobiology studies and therapy development for rare diseases; several have been generated recently from osteopetrotic patients bearing different mutations in *TCIRG1* [11, 12]. Ex vivo generation of genetically modified macrophages from human iPSCs has been proven feasible [13, 14]. Generation of functional osteoclasts using gene-corrected iPSCs derived from osteopetrotic mice has been demonstrated [15]. Similarly, a recent study generated functional osteoclasts using human iPSCs by targeting the fibroblasts of an osteopetrosis patient [16].

In this study, we used a lentiviral vector equipped with a ubiquitous chromatin opening element (CBX3-UCOE) [17] to express the functional human *TCIRG1* cDNA in iPSCs derived from an IMO patient with a homozygous c.11279G>A (IVS18+1) mutation in *TCIRG1*, and we show rescue of the resorptive function of iPSC-derived osteoclasts.

## Methods

### Derivation of fibroblasts from skin biopsy

A skin biopsy was obtained from a 2-year-old female with infantile malignant osteopetrosis (IMO). The biopsy was cut into small pieces and cultured on gelatin-coated 6-well culture dishes under coverslips in fibroblast medium (Dulbecco’s modified Eagle’s medium (DMEM) with 10% fetal bovine serum (FBS) and 2 mM GlutaMAX (Life Technologies)) supplemented with 4 ng/ml basic fibroblast growth factor (bFGF; PeproTech) for approximately 3 weeks before passaging.

### Reprogramming

Passage 1 and 2 IMO fibroblasts were used for IMO iPSC reprogramming, and the CCD-1100Sk human fibroblast cell line (ATCC® CRL-2211™) was used to generate control (CTRL) iPSCs. Reprogramming was performed with the CytoTune™ iPS 2.0 Sendai reprogramming kit (Invitrogen) following the manufacturer’s instructions. In brief, 5 × 10^4^, 10 × 10^4^, and 20 × 10^4^ cells were seeded into each well of a 6-well plate and cultured in fibroblast medium. Cells were transduced with the CytoTune™ iPS 2.0 Sendai reprogramming vector with multiplicities of infection (MOIs) of 5, 5, 5, and 3 (i.e., *OCT3/4* MOI = 5, *CMYC* MOI = 5, *SOX2* MOI = 5, *KLF* MOI = 3) 2 days after seeding (day 0) and incubated overnight. Reprogramming vectors were removed with a change to fresh medium the following day. Medium change was performed every other day until day 7. Transduced cells were replated onto murine embryonic fibroblast (MEF) culture dishes, and the medium was changed to iPSC medium (DMEM/F-12 with 20% knockout serum replacement, 0.1 nmol/l β-mercaptoethanol, 1 mmol/l l-glutamine, and 1% non-essential amino acids (Life Technologies)) supplemented with 10 ng/ml basic fibroblast growth factor (bFGF; PeproTech). iPSC medium was changed every day, and iPSC colonies appeared after 3 weeks of culture. Individual colonies were picked using pipette tips and expanded in iPSC medium on MEF culture dishes. In total, 12 colonies were picked for CTRL iPSC lines and 18 colonies for IMO iPSC lines.

Confirmation of the absence of the CytoTune™ iPS 2.0 Sendai reprogramming vectors was obtained by reverse transcription polymerase chain reaction (RT-PCR). Total RNA was extracted between P11 and P16 from both control and IMO lines using the RNeasy micro kit (Qiagen). Reverse transcription (RT) reactions were performed with SuperScript III (Invitrogen). Polymerase chain reaction (PCR) was performed following the instructions from the CytoTune™ iPS 2.0 Sendai reprogramming kit to detect the absence of SeV, *OCT3/4*, *KLF*, *SOX2*, and *CMYC*.

In total, 5 iPSC lines each from control and IMO reprogramming were obtained. After the initial screening, control 7 and IMO 1 iPSC lines were selected for further analyses based on their differentiation ability. Karyotyping was performed at P14–P16 and showed normal chromosome number and structure.

### Identification of the patient TCIRG1 mutation

Genomic DNA was extracted from P20 of both control and IMO iPSC lines using the DNeasy blood & tissue kit (Qiagen). The DNA fragment containing the mutation was amplified by PCR reaction with specific primers [18] (Table 1). PCR products were cleaned and sequenced. Sequence analyses were performed using the SnapGene software.

**Table 1:** Primer list [18, 19]

Target	Product size (bp)	Primer sequence
Sendai vector detection		
SeV	181	Forward GGATCACTAGGTGATATCGAGC
		Reverse ACCAGACAAGAGTTTAAGAGATATGTATC
*KLF4*	410	Forward TTCCTGCATGCCAGAGGAGCCC
		Reverse AATGTATCGAAGGTGCTCAA
*CMYC*	532	Forward TAACTGACTAGCAGGCTTGTCG
		Reverse TCCACATACAGTCCTGGATGATGATG
*SOX2*	451	Forward ATGCACCGCTACGACGTGAGCGC
		Reverse AATGTATCGAAGGTGCTCAA
*OCT4*	483	Forward CCCGAAAGAGAAAGCGAACCAG
		Reverse AATGTATCGAAGGTGCTCAA
Pluripotency and differentiation markers		
*NANOG*	237	Forward AAGGTCCCGGTCAAGAAACAG
		Reverse CTTCTGCGTCACACCATTGC
*BRACHYUR*	252	Forward TAAGGTGGATCTTCAGGTAGC
		Reverse CATCTCATTGGTGAGCTCCCT
*GOOSECOID*	89	Forward AACGCGGAGAAGTGGAACAAG
		Reverse CTGTCCGAGTCCAAATCGC
*MIXL1*	67	Forward CTGTTCCCCTCTCTCTGAAGA
		Reverse GGCAGAAAAGATGTGTTCCTCC
*FOXA2*	83	Forward GGAGCAGCTACTATGCAGAGC
		Reverse CGTGTTCATGCCGTTCATCC
*SOX17*	94	Forward GTGGACCGCACGGAATTTG
		Reverse GGAGATTCACACCGGAGTCA
*NESTIN*	145	Forward TTGCCTGCTACCCTTGAGAC
		Reverse GGGCTCTGATCTCTGCATCTAC
*SOX1*	287	Forward CAGTACAGCCCCATCTCCAAC
		Reverse GCGGGCAAGTACATGCTGA
*NEUROD*	523	Forward GCCCCAGGGTTATGAGACTATCACT
		Reverse CCGACAGAGCCCAGATGTAGTTCTT
*OTX2*	98	Forward TGTAGAAGCTATTTTTGTGGGTGA
		Reverse GAGCATCGTTCCATCTAACTTTTT
*PAX6*	162	Forward TGTCCAACGGATGTGTGAGT
		Reverse TTTCCCAAGCAAAGATGGAC
IMO mutation detection		
Human *TCIRG1 Exons 16–20*	1255	Forward GGTTCCTTTGCAGGTGTGCA
		Reverse CCTCTCCTGCCTCAGAGGTC

### Culture of iPSCs

Human iPSCs were cultured in mTeSR medium (STEMCELL Technologies) on 6-well plates coated with Matrigel (STEMCELL Technologies). Cells were passaged every 5–7 days with 1 mg/ml dispase (STEMCELL Technologies) at a ratio of 1:6 to 1:8.

### Immunostaining of pluripotency markers

iPSCs were seeded on 12-well chamber slides (Ibidi, 81201) and cultured for 2 days. Cells were then rinsed with Dulbecco’s phosphate-buffered saline (DPBS) and fixed with ice-cold 4% paraformaldehyde (PFA) for 15 min. Cells were then incubated in 5% normal serum and 1% Triton X-100 in PBS for 45 min at room temperature. Primary antibodies were diluted in 5% normal serum (Merck) and 1% Triton X-100, and incubation was carried out overnight at 4 °C. Primary antibodies were detected by incubating with appropriate fluorescent secondary antibodies for 90 min at room temperature. For nuclear staining, 10 mg/ml Hoechst 33342 (Thermo Fisher Scientific) was added for 10 min after the incubation with secondary antibodies. For labeling TRA-1-60 membrane protein, cells were first incubated in 5% normal serum and 0.025% Triton X-100 for 45 min, then stained with mouse anti-TRA-1-60 IgM diluted in 5% normal serum and 0.025% Triton X-100 overnight at 4 °C. The secondary antibody was added for 90 min at room temperature. For double labeling with other primary antibodies, cells were fixed again with ice-cold PFA for 10 min, and staining was performed as described above. Images were obtained using a Zeiss 780 laser confocal microscope (Zeiss).

### Examination of pluripotency by in vitro differentiation into three germ layers

Undirected differentiation was performed using a previously published protocol [19]. Briefly, embryoid body (EB) formation was achieved using collagenase IV treatment. EBs were cultured in DMEM Low Glucose supplemented with 10% FBS and 2 mM l-glutamine for 10 days. The medium was changed every other day. Total RNA was purified from EBs on day 10 and used in RT reactions with SuperScript III (Invitrogen). One microliter of RT product was used for PCR to detect the gene expression from three germ layers: *NANOG* was used to test for pluripotency; *BRACHYUR*, *GOOSECOID*, and *MIXL1* for mesoderm; *FOXA2*, *SRY*, and *SOX17* for endoderm; and *NESTIN*, *SOX1*, *NEUROD*, *OTX2*, and *PAX6* for ectoderm. Primers used for PCR are listed in Table 1.

### Cloning of the human TCIRG1 construct

**Fig. 1:**
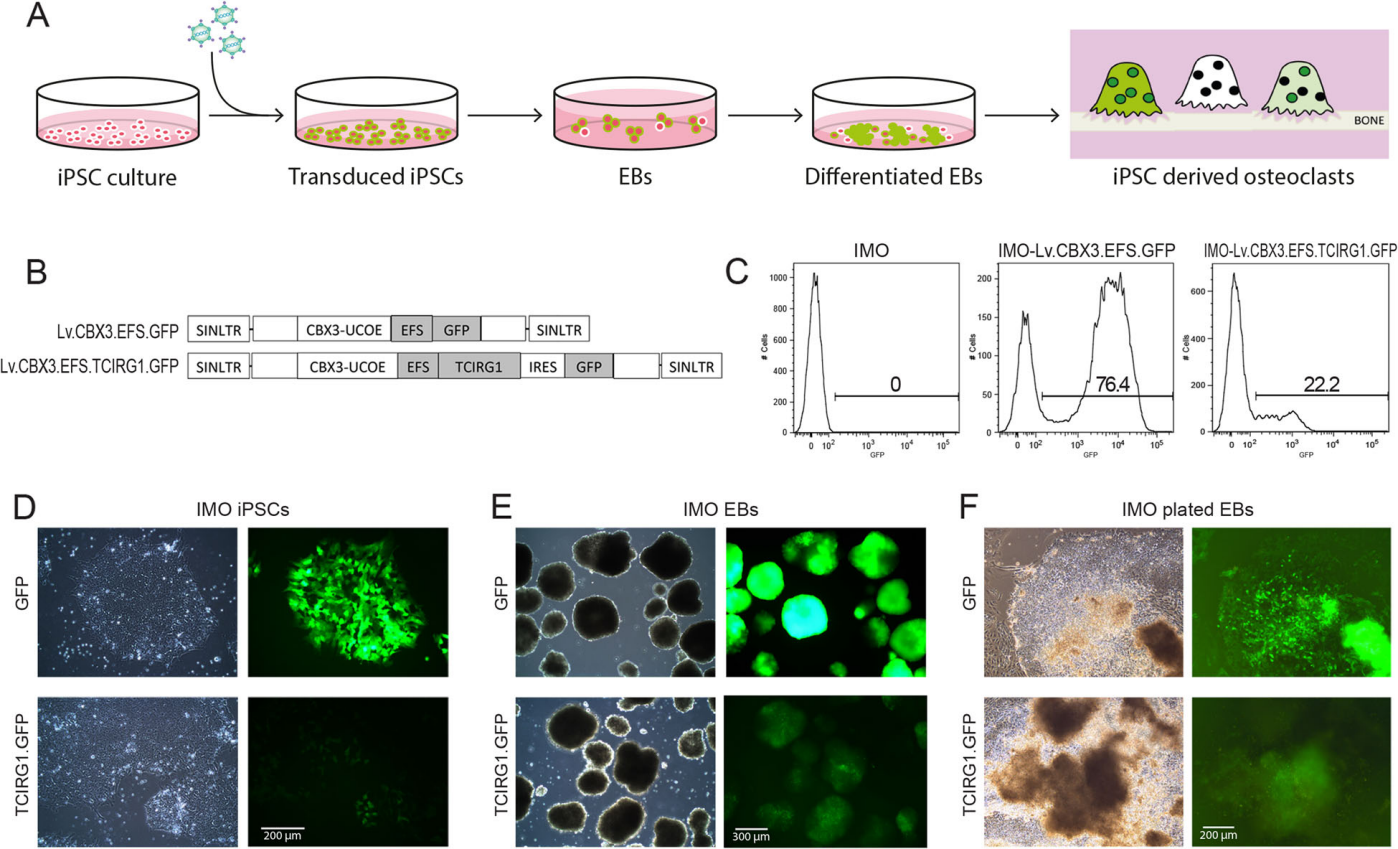
Lentiviral-mediated gene expression in IMO iPSC-derived monocytes. Schematic representation of the experimental design (**a**). IMO iPSCs were transduced with a lentiviral vector expressing *TCIRG1* and containing the CBX3-UCOE element (**b**). EBs were generated from the iPSCs, transferred to tissue culture plates after 4 days of culture, and differentiated in X-VIVO media supplemented with M-CSF and IL-3. The presence of GFP^+^ cells was verified throughout the culture at iPSC stage (**c**, **d**), at EB stage (**e**), and during EB differentiation (**f**)

Human full-length *TCIRG1* cDNA was cloned into a lentiviral backbone under the control of the CBX3-EFS promoter (the pRRL.PPT.CBX3.EFS.GFP.PRE backbone was a generous gift from Dr. Dirk Hoffmann). The EGFP fragment was replaced by *hTCIRG1* cDNA using BamHI and SalI restriction cloning. Internal ribosomal entry site (IRES)-GFP was inserted after h*TCIRG1* by SalI restriction cloning to visualize the transgene expression (Fig. 1b).

### Lentiviral vector production

Lentiviral vectors were produced by transient transfection of HEK293T cells through calcium phosphate precipitation as described previously [20]. Lentiviral vectors from the supernatant were collected 48 h and 72 h post-transfection. The virus was filtered and concentrated by ultracentrifugation. Viral titers were determined by flow cytometry using the GFP signal in transduced HT1080 cells.

### Transduction of iPSCs

1 × 10^5^ iPSCs were individualized with TrypLE Select (Invitrogen) and seeded the day before transduction on a Matrigel-coated (STEMCELL Technologies) 12-well plate. The following day, cells were counted and lentiviral transduction was performed at MOIs of 20–100 for 6 h in 400 μl iPSC medium before medium change.

### Monocyte differentiation of human iPSCs

Monocyte differentiation was performed using a previously published protocol [14]. In brief, control and IMO iPSC colonies were treated with dispase (0.5 mg/ml) for EB formation. EBs were cultured in iPSC medium for 4 days on low attachment dishes. At day 4, EBs were transferred onto 6-well plates with approximately 50 EBs/well in monocyte differentiation medium (X-VIVO™ 15, Lonza), supplemented with 100 ng/ml human M-CSF (R&D), 25 ng/ml human IL-3 (PeproTech), 2 mM GlutaMAX (Invitrogen), 100 U/ml penicillin, 100 μg/ml streptomycin (Invitrogen), and 0.055 mmol/l β-mercaptoethanol (Invitrogen). Medium change was performed every 5 days, and monocytes were harvested once a week from week 5 onward (Fig. 1a).

### Flow cytometry

Control and IMO iPSCs were harvested using TrypLE Select treatment and suspended in 2% FBS in PBS. Cells were stained with 4′,6-diamidino-2-phenylindole (DAPI), and the expression of GFP was detected with the BD Canto (Becton & Dickinson). The results were analyzed using the FlowJo software.

### Osteoclastogenesis

The non-adherent cell fraction was harvested weekly from the differentiating EB cultures and reseeded onto 96-well plates on bovine cortical bone slices at a density of 1 × 10^5^/well for cell assays and onto 24-well plates on plastic at 5 × 10^5^/well for western blot. The cells were incubated at 37 °C and 5% CO_2_ in αMEM (Gibco) containing 10% fetal bovine serum (Sigma-Aldrich), 100 U/ml penicillin (Sigma-Aldrich), 100 μg/ml streptomycin (Sigma-Aldrich), and 388 μg/l thymidine (Sigma-Aldrich) for 3 days in the presence of 50 ng/ml human M-CSF (R&D Systems) and then further differentiated for an additional 10 days in the presence of 50 ng/ml human M-CSF and 50 ng/ml human RANKL (R&D Systems) with medium changes every 2–3 days. After 13 days, the cells were either fixed in 4% formaldehyde for further analyses or lysed for western blot analysis.

### Western blot

Osteoclasts differentiated from both control and IMO iPSCs were collected and lysed with RIPA buffer (Thermo Fisher Scientific) supplemented with Halt Protease Inhibitor Cocktail (Thermo Fisher Scientific) following the manufacturer’s instructions. Ten microliters of cell lysate was mixed with 10 μl of 2× sample buffer (Thermo Fisher Scientific) and heated at 70 °C for 10 min. Samples were loaded on Bolt™ 4–12% Bis-Tris gel (Invitrogen) together with MagicMark (Thermo Fisher Scientific) and run for 25 min at 200 V. Proteins were transferred to PVDF membranes using an iBlot 2 machine (Thermo Fisher Scientific). The membrane was blocked with 5% blocking reagent (GE Healthcare) in PBS with Tween 20 (Thermo Fisher Scientific) for 45 min at room temperature before incubation with primary (anti-TCIRG1 or anti-p38 as a loading control) or secondary antibodies overnight at 4 °C. Antibodies used for western blot are listed in Table 2. Images were taken with the GelDoc system (Bio-Rad).

**Table 2:** Antibody list

Antibodies	Catalog number	Company	Dilution
Immunostaining			
Mouse anti-oct3/4	sc5279	Santa Cruz	1:50
Rabbit anti-Nanog	ab21624	Abcam	1:150
Mouse anti-TRA-1-60	MAB4360	Merck	1:100
Rabbit anti-SOX2	AB5603	Merck	1:500
Alexa Fluor 488 Donkey anti-mouse IgG (H+L)	A21202	Thermo Fisher Scientific	1:2000
Alexa Fluor 568 Donkey anti-rabbit IgG (H+L)	A10042	Thermo Fisher Scientific	1:2000
Alexa Fluor 647 Donkey anti-rabbit IgG (H+L)	A31573	Thermo Fisher Scientific	1:2000
Western blot			
Mouse anti-human TCIRG1	H00010312	Abnova	1:1000
Rabbit anti-p38 MAPK	9212	Cell Signaling Technology	1:1000
ECL Mouse IgG, HRP-linked whole Ab (from sheep)	NA9310	GE Healthcare	1:5000
ECL Rabbit IgG, HRP-linked *F*(*ab*′)_2_ fragment (from donkeys)	NA9340	GE Healthcare	1:5000

### TRAP activity measurements

One to 20 μl of media from 96-well cell cultures were added to a 96-well plate and diluted with water to a volume of 20 μl. The diluted samples were incubated with 80 μl freshly prepared reaction buffer (0.25 mol/l acetic acid, 0.125% Triton X-100, 0.25 mol/l NaCl, 2.5 mmol/l EDTA, 1.1 mg/ml of ascorbic acid, 5.75 mg/ml of disodium tartrate, 2.25 mg/ml of 4-nitrophenylphosphate, pH 5.5) at 37 °C for 1 h in the dark, and the reaction was then stopped by adding 100 μl of 0.3 mol/l NaOH. Absorbance was measured at 405 nm with 650 nm as a reference.

### CTX-I release

The release of the c-terminal type I collagen fragments (CTX-I) from resorbed bone slices was determined using the CrossLaps for Culture kit (IDS, The Boldons, UK), according to the manufacturer’s instructions.

### TRAP staining

Osteoclasts were fixed in 4% formaldehyde for 20 min and stained for tartrate-resistant acid phosphatase (TRAP) using the leukocyte acid phosphatase kit (Sigma-Aldrich). Digital micrographs were obtained using a × 40 objective and an Olympus digital camera mounted on an Olympus BX43 microscope using the Image View software.

### Resorption pit formation

Resorption pits on the fixed bone slices were visualized after washing them with water, removing the remaining cells by lysing them with RIPA buffer and scrubbing with a cotton swab followed by staining with hematoxylin for 7 min. Excess dye was removed by scrubbing the bones with a cotton swab. Digital micrographs were obtained using a × 10 objective and an Olympus digital camera mounted on an Olympus BX43 microscope using the Image View software.

## Results

### Lentiviral-mediated gene expression in IMO iPSC-derived monocytes

iPSCs were generated from a 2-year-old female IMO patient carrying a homozygous c.11279G>A (IVS18+1) mutation in *TCIRG1*. Fibroblasts were isolated from a skin biopsy and reprogrammed using integration-free Sendai viral vectors. CTRL iPSCs were generated from a normal fibroblast cell line. Characterization confirmed that the generated iPSC lines expressed pluripotency markers, retained the disease-causing mutation, did not retain the reprogramming vector, and showed pluripotent differentiation capacity (Suppl Fig. 1). IMO iPSCs were transduced with a lentiviral vector expressing *TCIRG1* and GFP under the elongation factor 1 alpha short (EFS) promoter and containing the CBX3-UCOE element (Lv.CBX3.EFS.TCIRG1.GFP) in order to prevent silencing of the transgene expression upon differentiation (Fig. 1a, b). Both IMO iPSCs and CTRL iPSCs were transduced with the control vector expressing only GFP (Lv.CBX3.EFS.GFP) (Fig. 1b). Transduction efficiency was measured by flow cytometry 3 days after transduction, and a GFP^+^ cell population was clearly detected in the cells transduced with either vector but the efficiency was higher with the control vector (Fig. 1c). Differentiation of the iPS cells to monocytes was performed according to a previously described protocol [14]. EBs were generated from the iPSCs, transferred to tissue culture plates after 4 days, and differentiated in X-VIVO media supplemented with M-CSF and IL-3 (Fig. 1a). The presence of GFP^+^ cells was verified by fluorescence microscopy throughout the culture at the iPSC stage (Fig. 1d), at the EB stage (Fig. 1e) and during differentiation of the plated EBs (Fig. 1f) indicating stable transgene expression.

### Restored resorptive function in osteoclasts generated from patient-derived IMO iPSCs after TCIRG1 gene transfer

**Fig. 2:**
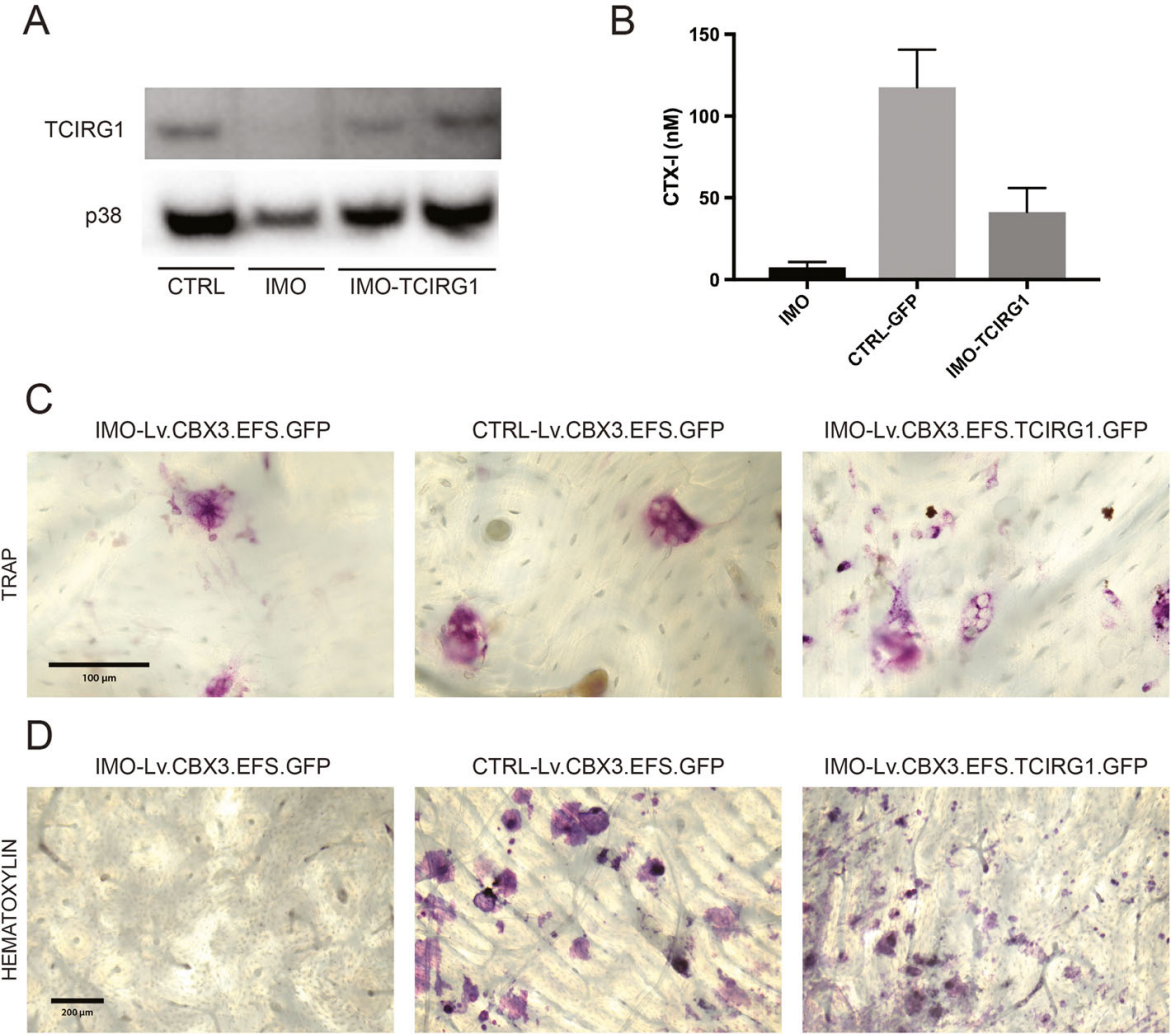
Restored resorptive function in osteoclasts generated from patient-derived IMO iPSCs after *TCIRG1* gene transfer. Non-adherent cells were harvested weekly from week 5 onward and differentiated into osteoclasts on plastic or bone slices for 13 days in the presence of M-CSF and RANKL. Western blot analysis was performed on cell lysates from mature osteoclasts (**a**). The concentration of CTX-I (**b**) was measured in the media at day 13. Data are shown as the mean ± SEM, *n* = 4. Bone slices were stained for TRAP^+^ cells (**c**) and then for resorption pits (**d**) after cell lysis. Images represent bone slices after culture of IMO-GFP, CTRL-GFP, and IMO-TCIRG1 iPSC-derived osteoclasts

The non-adherent cells from differentiating EB cultures were harvested weekly from week 5 onward and differentiated into osteoclasts on plastic for western blot analysis. TCIRG1 protein was expressed in the mature osteoclasts derived from transduced cells at day 13 of culture (Fig. 2a). iPSC-derived monocytes were also differentiated into osteoclasts on bone slices, and this was confirmed by assessing the TRAP activity in the culture medium whereas their ability to resorb bone was evaluated by measuring the levels of CTX-I in the medium. TRAP activity was present in the media of all osteoclast cultures (data not shown). A 5-fold increase in CTX-I levels was observed in IMO iPSC-derived osteoclasts transduced with the rescue vector Lv.CBX3.EFS.TCIRG1.GFP compared to non-transduced IMO iPSC-derived osteoclasts (Fig. 2b), indicating an increase in resorptive activity and at least partial restoration of function. Based on the CTX-I levels, the resorption of rescued IMO osteoclasts was 35% of that of osteoclasts derived from CTRL iPSCs (Fig. 2b). After 13 days of differentiation, TRAP^+^ multinucleated osteoclasts were present in all conditions (Fig. 2c) and the IMO osteoclasts generated from iPSCs transduced with Lv.CBX3.EFS.TCIRG1.GFP had formed a high number of clearly visible pits, whereas the bone slices with non-transduced IMO osteoclasts were almost free of pits (Fig. 2d) as expected.

## Discussion

Human iPSCs from healthy fibroblasts have been shown capable of differentiating into monocytes with the expression of pre-osteoclast markers in the presence of specific cytokines [14]. Furthermore, iPSCs subjected to transduction with lentiviral vectors [14] or with lentiviral vectors equipped with a CBX3-UCOE element [13] have been shown to retain high levels of marker gene expression after macrophage differentiation.

By applying the differentiation protocol mentioned above to iPSCs from normal fibroblasts and from fibroblasts of an IMO patient, we were able to obtain high amounts of cells that after further differentiation with M-CSF and RANKL exhibited the TRAP^+^ multinucleated phenotype typical of osteoclasts. Osteoclasts derived from normal iPSCs were capable of resorbing bone effectively in vitro while osteoclasts from IMO patient iPSCs exhibited strongly impaired resorption as expected.

For the gene transfer experiments, we chose a self-inactivating (SIN) lentiviral vector with a CBX3-UCOE element. When iPSCs were transduced with the control vector (Lv.CBX3.EFS.GFP) or the rescue vector (Lv.CBX3.EFS.TCIRG1.GFP) and then differentiated into monocytes, the presence of efficiently transduced cells was verified at different culture stages, even though GFP intensity was lower with the vector expressing *TCIRG1* most likely due to the size of the bicistronic vector and the presence of the IRES. Upon differentiation of transduced monocytes to mature osteoclasts, TCIRG1 protein was present in the rescued cells indicating stable gene expression. Transducing at the iPSC stage without clonal selection allows for a more accurate assessment of the level of correction in the subsequent functionality assays, as compared to the transduction of fibroblasts [16] followed by reprogramming where differences between the iPSC clones must be considered.

The main objective of the current work was to assess the functional restoration of osteoclasts differentiated from the transduced cells. The ability of osteoclasts to resorb bone was evaluated by measuring the release of the resorption marker CTX-I into the media. Resorption of osteoclasts derived from IMO iPSCs transduced with the rescue vector was on average 35% that of osteoclasts derived from normal iPSCs based on CTX-I. As further confirmation of the rescued phenotype, mature IMO osteoclasts transduced with Lv.CBX3.EFS.TCIRG1.GFP formed clearly visible resorption pits when differentiated on bone slices whereas pits were in most cases completely absent in IMO controls. This clearly establishes that the introduction of non-mutated *TCIRG1* into IMO iPSCs and thus the resulting osteoclasts restore their osteoclastic ability to acidify the resorption lacunae and resorb the bone in vitro.

Due to the lack of a mouse model in which functionality of human osteoclasts can be tested, we cannot determine directly if the partial restoration of osteoclast function would translate into a complete rescue of the bone phenotype in vivo; however, there are indications that it could be sufficient. Coccia et al. after transplantation of a 5-month-old girl with IMO with bone marrow from her brother used fluorescent in situ hybridization (FISH) for the Y chromosome to study donor chimerism and found that only 30% of mononuclear cells in the blood were of male origin [21]. Despite this low level of chimerism, the transplant was deemed clinically successful. Furthermore, in a previous work, we showed that the in vitro bone resorption capacity of osteoclasts formed from bone marrow cells of transplanted *oc/oc* mice was only 14% of controls, but despite this low level of in vitro resorption, osteopetrosis was rapidly reversed in vivo [22]. This indicates that only a small part of the resorptive potential of the total osteoclast population is needed and used in vivo.

The mutational spectrum causing IMO is wide and complex [18]; generation of new patient-derived iPSCs and their differentiation to mature osteoclasts will contribute to the resources available for the study of disease mechanisms. Furthermore, the rescue of the phenotype of IMO osteoclasts by gene transfer of the *TCIRG1* cDNA to patient iPSCs has important implications for the development of new therapies for this disease. In our previous studies, we have provided proof of concept for an autologous approach to treating osteopetrosis by hematopoietic stem cell-targeted gene therapy with clinically applicable lentiviral vectors in IMO patient CD34^+^ peripheral blood cells [23] and in the oc/oc osteopetrotic mouse model [9]. However, it has been proposed that IMO patients could also benefit from early transplantation of myeloid progenitors differentiated towards the osteoclast lineage [24]. For this purpose, autologous iPSCs could potentially provide an unlimited source of pre-osteoclasts to support the early phase of recovery after autologous gene therapy with corrected stem cells.

## Conclusions

Non-resorbing osteoclasts were generated from an IMO patient-derived iPSC line, and the phenotype was partially rescued through lentiviral-mediated gene transfer, providing a valuable resource for pathobiology studies and therapy development for this form of severe osteopetrosis.

## Supplementary information

**Additional file 1:Supplementary Figure 1.** Characterization of the generated iPSC lines (IMO and CTRL). Immunofluorescent staining (A) demonstrated that IMO iPSCs expressed pluripotency markers at the protein level. The presence of the patient mutation (B) was verified in the IMO iPSCs and was absent in the CTRL iPSCs. The absence of the reprogramming vector (C) was confirmed for all the iPSC lines. RT-PCR of markers for pluripotency and the three germ layers (D) was performed.

## Data Availability

All data generated or analyzed during this study are included in this published article and its supplementary information file.

## Abbreviations

bFGF: Basic fibroblast growth factor
CBX3-UCOE: Ubiquitous chromatin opening element
cDNA: Complementary DNA
CTRL: Control
CTX-I: c-terminal type I collagen fragment
DAPI: 4′,6-Diamidino-2-phenylindole
DMEM: Dulbecco’s modified Eagle’s medium
EB: Embryoid body
EDTA: Ethylenediaminetetraacetic acid
EFS: Elongation factor 1 alpha short
FBS: Fetal bovine serum
FISH: Fluorescent in situ hybridization
GFP: Green fluorescent protein
HSC: Hematopoietic stem cell
HSCT: Hematopoietic stem cell transplantation
IL-3: Interleukine-3
IMO: Infantile malignant osteopetrosis
iPSCs: Induced pluripotent stem cells
IRES: Internal ribosomal entry site
Lv: Lentiviral vector
M-CSF: Macrophage colony-stimulating factor
MEF: Murine embryonic fibroblasts
MOI: Multiplicity of infection
PBS: Phosphate-buffered saline
PCR: Polymerase chain reaction
PFA: Paraformaldehyde
RANKL: Receptor activator of nuclear factor kappa-Β ligand
RIPA: Radioimmunoprecipitation assay
RT: Reverse transcription
SEM: Standard error of the mean
SIN: Self-inactivating
TRAP: Tartrate-resistant acid phosphatase

## References

[1] Sobacchi C, Schulz A, Coxon FP, Villa A, Helfrich MH (2013). Osteopetrosis: genetics, treatment and new insights into osteoclast function. Nat Rev Endocrinol.

[2] Wu CC, Econs MJ, DiMeglio LA, Insogna KL, Levine MA, Orchard PJ (2017). Diagnosis and management of osteopetrosis: consensus guidelines from the Osteopetrosis Working Group. J Clin Endocrinol Metab.

[3] Fasth A, Porras O (1999). Human malignant osteopetrosis: pathophysiology, management and the role of bone marrow transplantation. Pediatr Transplant.

[4] Wilson CJ, Vellodi A (2000). Autosomal recessive osteopetrosis: diagnosis, management, and outcome. Arch Dis Child.

[5] Del Fattore A, Cappariello A, Teti A (2008). Genetics, pathogenesis and complications of osteopetrosis. Bone..

[6] Schulz AS, Classen CF, Mihatsch WA, Sigl-Kraetzig M, Wiesneth M, Debatin KM (2002). HLA-haploidentical blood progenitor cell transplantation in osteopetrosis. Blood..

[7] Shadur B, Zaidman I, NaserEddin A, Lokshin E, Hussein F, Oron HC (2018). Successful hematopoietic stem cell transplantation for osteopetrosis using reduced intensity conditioning. Pediatr Blood Cancer.

[8] Johansson MK, de Vries TJ, Schoenmaker T, Ehinger M, Brun AC, Fasth A (2007). Hematopoietic stem cell-targeted neonatal gene therapy reverses lethally progressive osteopetrosis in oc/oc mice. Blood..

[9] Lofvall H, Rothe M, Schambach A, Henriksen K, Richter J, Moscatelli I (2019). Hematopoietic stem cell-targeted neonatal gene therapy with a clinically applicable lentiviral vector corrects osteopetrosis in oc/oc mice. Hum Gene Ther.

[10] Askmyr M, Flores C, Fasth A, Richter J (2009). Prospects for gene therapy of osteopetrosis. Curr Gene Ther.

[11] Lanzi G, Ferraro RM, Masneri S, Piovani G, Barisani C, Sobacchi C (2019). Generation of 3 clones of induced pluripotent stem cells (iPSCs) from a patient affected by autosomal recessive osteopetrosis due to mutations in TCIRG1 gene. Stem Cell Res.

[12] Okur FV, Cevher I, Ozdemir C, Kocaefe C, Cetinkaya DU (2019). Osteopetrotic induced pluripotent stem cells derived from patients with different disease-associated mutations by non-integrating reprogramming methods. Stem Cell Res Ther.

[13] Ackermann M, Kuhn A, Kunkiel J, Merkert S, Martin U, Moritz T (2017). Ex vivo generation of genetically modified macrophages from human induced pluripotent stem cells. Transfus Med Hemother.

[14] van Wilgenburg B, Browne C, Vowles J, Cowley SA (2013). Efficient, long term production of monocyte-derived macrophages from human pluripotent stem cells under partly-defined and fully-defined conditions. PLoS One.

[15] Neri T, Muggeo S, Paulis M, Caldana ME, Crisafulli L, Strina D (2015). Targeted gene correction in osteopetrotic-induced pluripotent stem cells for the generation of functional osteoclasts. Stem Cell Rep.

[16] Chen W, Twaroski K, Eide C, Riddle MJ, Orchard PJ, Tolar J (2019). TCIRG1 transgenic rescue of osteoclast function using induced pluripotent stem cells derived from patients with infantile malignant autosomal recessive osteopetrosis. J Bone Joint Surg Am.

[17] Muller-Kuller U, Ackermann M, Kolodziej S, Brendel C, Fritsch J, Lachmann N (2015). A minimal ubiquitous chromatin opening element (UCOE) effectively prevents silencing of juxtaposed heterologous promoters by epigenetic remodeling in multipotent and pluripotent stem cells. Nucleic Acids Res.

[18] Sobacchi C, Frattini A, Orchard P, Porras O, Tezcan I, Andolina M (2001). The mutational spectrum of human malignant autosomal recessive osteopetrosis. Hum Mol Genet.

[19] Hildebrand L, Rossbach B, Kuhnen P, Gossen M, Kurtz A, Reinke P (2016). Generation of integration free induced pluripotent stem cells from fibrodysplasia ossificans progressiva (FOP) patients from urine samples. Stem Cell Res.

[20] Hamaguchi I, Woods NB, Panagopoulos I, Andersson E, Mikkola H, Fahlman C (2000). Lentivirus vector gene expression during ES cell-derived hematopoietic development in vitro. J Virol.

[21] Coccia PF, Krivit W, Cervenka J, Clawson C, Kersey JH, Kim TH (1980). Successful bone-marrow transplantation for infantile malignant osteopetrosis. N Engl J Med.

[22] Flores C, de Vries TJ, Moscatelli I, Askmyr M, Schoenmaker T, Langenbach GE (2010). Nonablative neonatal bone marrow transplantation rapidly reverses severe murine osteopetrosis despite low-level engraftment and lack of selective expansion of the osteoclastic lineage. J Bone Miner Res.

[23] Moscatelli I, Lofvall H, Schneider Thudium C, Rothe M, Montano C, Kertesz Z (2018). Targeting NSG mice engrafting cells with a clinically applicable lentiviral vector corrects osteoclasts in infantile malignant osteopetrosis. Hum Gene Ther.

[24] Cappariello A, Berardi AC, Peruzzi B, Del Fattore A, Ugazio A, Bottazzo GF (2010). Committed osteoclast precursors colonize the bone and improve the phenotype of a mouse model of autosomal recessive osteopetrosis. J Bone Miner Res.

