# Supplementary figures and images for: Generation of gene-corrected functional osteoclasts from osteopetrotic induced pluripotent stem cells
Source: Stem Cell Res Ther. 2020 May 15;11:179. doi: 10.1186/s13287-020-01701-y (PMC7227215; doi:10.1186/s13287-020-01701-y)

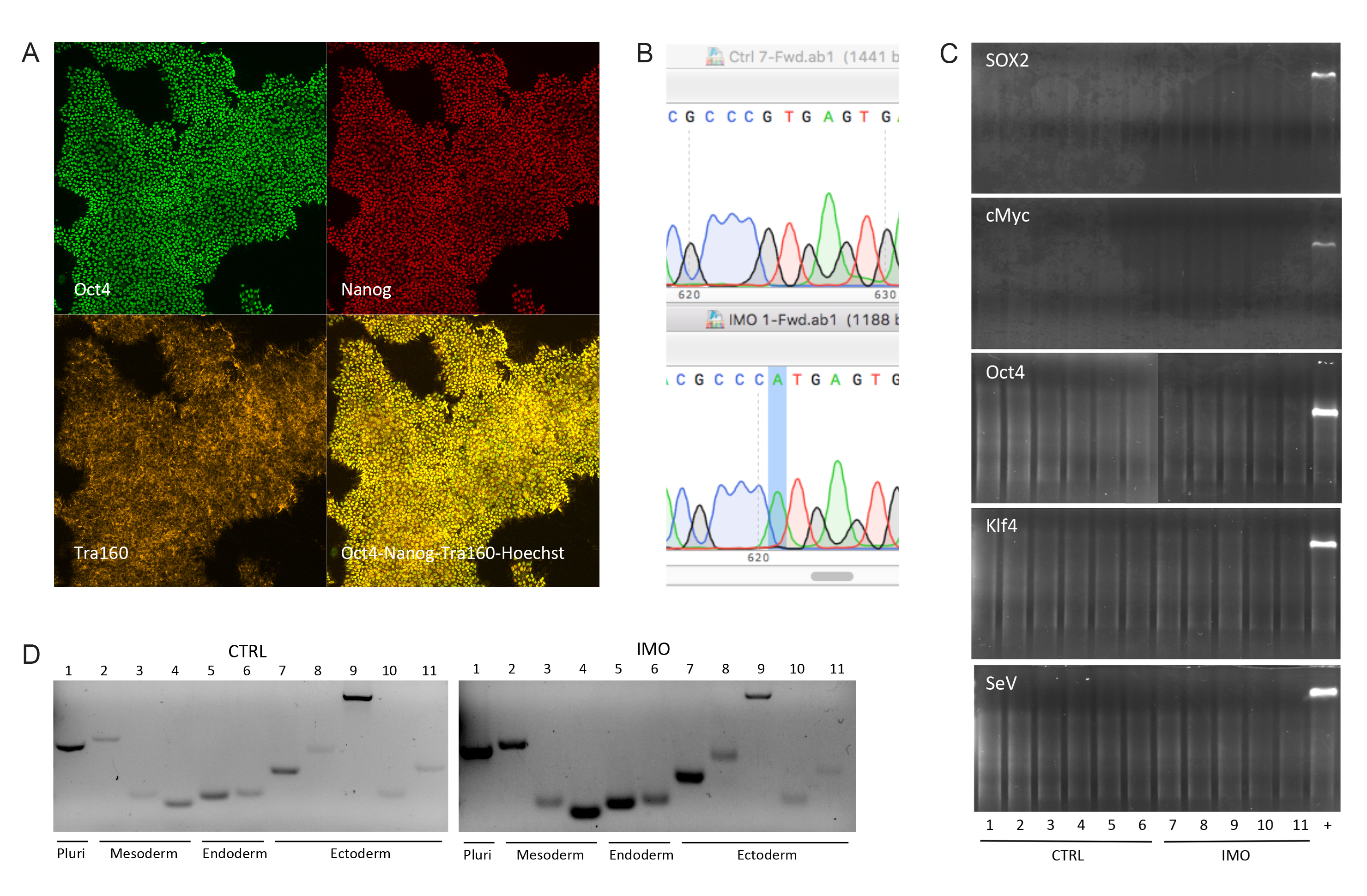

Supplement: Supplementary file 1 — Additional file 1:Supplementary Figure 1. Characterization of the generated iPSC lines (IMO and CTRL). Immunofluorescent staining (A) demonstrated that IMO iPSCs expressed pluripotency markers at the protein level. The presence of the patient mutation (B) was verified in the IMO iPSCs and was absent in the CTRL iPSCs. The absence of the reprogramming vector (C) was confirmed for all the iPSC lines. RT-PCR of markers for pluripotency and the three germ layers (D) was performed. [file 13287_2020_1701_MOESM1_ESM.jpg]
